# Supplementary material for: A concerted neuron–astrocyte program declines in ageing and schizophrenia
Source: Nature. 2024 Mar 6;627(8004):604–11. doi: 10.1038/s41586-024-07109-5 (PMC10954558; doi:10.1038/s41586-024-07109-5)
Supplement: Supplementary file 1 — Supplementary Note (analyses related to SNAP in the genetics of schizophrenia), the legends for Supplementary Tables 1–9 and Supplementary Figs. 1–13. [file 41586_2024_7109_MOESM1_ESM.pdf]

nature portfolio

https://doi.org/10.1038/s41586-024-07109-5

Supplementary information

A concerted neuron–astrocyte program declines in ageing and schizophrenia

In the format provided by the authors and unedited

SUPPLEMENTARY INFORMATION

A concerted neuron-astrocyte program declines in ageing and schizophrenia

Emi Ling[1,2 §], James Nemesh[1,2], Melissa Goldman[1,2], Nolan Kamitaki[1,2,3], Nora Reed[1,2], Robert E. Handsaker[1,2], Giulio Genovese[1,2], Jonathan S. Vogelgsang[4,5], Sherif Gerges[1,2], Seva Kashin[1,2], Sulagna Ghosh[1,2], John M. Esposito[4], Kiely Morris[4], Daniel Meyer[1,2], Alyssa Lutservitz[1,2], Christopher D. Mullally[1,2], Alec Wysoker[1,2], Liv Spina[1,2], Anna Neumann[1,2], Marina Hogan[1,2], Kiku Ichihara[1,2], Sabina Berretta[1,4,5,6 * §], Steven A. McCarroll[1,2 * §]

1. Stanley Center for Psychiatric Research, Broad Institute of MIT and Harvard, Cambridge, MA, USA
2. Department of Genetics, Harvard Medical School, Boston, MA, USA
3. Department of Biomedical Informatics, Harvard Medical School, Boston, MA, USA
4. McLean Hospital, Belmont, MA, USA
5. Department of Psychiatry, Harvard Medical School, Boston, MA, USA
6. Program in Neuroscience, Harvard Medical School, Boston, MA, USA

* Jointly supervised this work

§ Correspondence: smccarro@broadinstitute.org, sberretta@mclean.harvard.edu, eling@broadinstitute.org

TABLE OF CONTENTS

Supplementary Note **1**
Synaptic Neuron-Astrocyte Program (SNAP) in the genetics of schizophrenia 1
Cell-identity gene expression and schizophrenia genetics: replication of earlier results 1
Cellular-program (SNAP) gene expression and genes implicated in schizophrenia 1
Cellular-program (SNAP) gene expression and schizophrenia genetics: genome-wide genetic association statistics 3
References 5

Supplementary Tables **6**

Supplementary Figures **7**
Supplementary Figure 1. Cell-type classification and composition analysis 8
Supplementary Figure 2. Expression of glutamatergic neuron-subtype marker genes 9
Supplementary Figure 3. Expression of GABAergic neuron-subtype marker genes 10
Supplementary Figure 4. Glutamatergic neuron-subtype composition analysis across donors 11
Supplementary Figure 5. GABAergic neuron-subtype composition analysis across donors 12
Supplementary Figure 6. Robustness of Latent Factor 4 (LF4) to analysis parameters 13
Supplementary Figure 7. Latent factor analysis of cerebrospinal fluid (CSF) proteomics data from different individuals identifies a factor resembling SNAP 14
Supplementary Figure 8. Concerted synaptic investments by neurons and astrocytes 15
Supplementary Figure 9. Identification of astrocyte transcriptional neighborhoods associated with schizophrenia case-control status by co-varying neighborhood analysis 16
Supplementary Figure 10. Expression across cell types of genes most strongly recruited by SNAP-a 17
Supplementary Figure 11. Expression across cell types of genes most strongly recruited by SNAP-n 18
Supplementary Figure 12. Heritability enrichment for 26 traits among the top 2,000 astrocyte-identity or astrocyte-activity (SNAP-a) genes 19
Supplementary Figure 13. Calculation of polygenic risk scores for schizophrenia 20

SUPPLEMENTARY NOTE

Synaptic Neuron-Astrocyte Program (SNAP) in the genetics of schizophrenia

Cell-identity gene expression and schizophrenia genetics: replication of earlier results

Many earlier studies [1–3] have found that genes most strongly expressed by neurons relative to other CNS cell types, but not genes most strongly expressed by astrocytes or other glia, are enriched for the genes implicated by human-genetic studies in schizophrenia. We first replicated these findings using the data from the current experiments. Genes that were preferentially expressed in neurons (as defined by the criteria used in the earlier studies) exhibited enrichment for schizophrenia-risk genes and alleles by a variety of analysis methods, but genes that were preferentially expressed in astrocytes did not.

For example, the following is an analysis of genes' schizophrenia association signals from common genetic variation (gene-level Z-scores, calculated using MAGMA) versus these sets of "cell-type preferentially expressed genes" (as defined by the methods of earlier work and applied to the current data), for neurons and astrocytes. For neurons, for example, this gene-set comprises the 2,000 genes for which neurons exhibit the highest quantitative expression levels relative to other cell types. As expected from earlier results, we see strong significance for neurons but not for astrocytes:

Linear regression of genes' levels of schizophrenia association (MAGMA z-scores) against their membership in cell-type-specific expression groups (top 2,000 genes)

	Estimate	Std. Error	t value	Pr(>|t|)
(Intercept)	1.05572978	0.01390269	75.937099	0.000000e+00
genes w/ expression most specific to neurons	0.16819618	0.03764630	4.467801	**7.954875e-06**
genes w/ expression most specific to astrocytes	0.05412946	0.03764630	1.437843	1.504975e-01

(Here we used a single, composite "neuronal" set of expression values for the analysis, but we had very similar results when we used specific types or subtypes of cortical neurons, reflecting the strong correlation among their gene-expression levels.)

Thus, these well-established and expected relationships are also visible in the current, human cell-type-specific expression data.

Cellular-program (SNAP) gene expression and genes implicated in schizophrenia

The above analysis, like other analyses to date, treats cell types as fixed levels of cell-identity gene expression, rather than as dynamic biological entities that utilize gene expression in ways whose variation is also meaningful. Cell-identity gene expression actually tells us little about SNAP-a: of the 500 genes most strongly recruited by SNAP-a, more than 90% are also robustly expressed in neurons and/or other glia of various types; less than half (203 of 500) are most strongly expressed in astrocytes, reflecting that biological functions such as synaptic adhesion and neurotransmitter uptake are also performed by neurons. Rather, it is the close

transcriptional co-regulation of these genes in astrocytes by SNAP-a that appears to strongly distinguish astrocytes from neurons **(Fig. 3k)**.

Cell types would ideally be considered, not only in terms of static cell-identity gene expression, but by their repertoires of *dynamic* transcriptional responses, such as SNAP-a, the set of astrocyte gene-expression changes that appear to be implemented in tandem with synaptic gene-expression changes in neurons (SNAP-n). To do so, we started with the 500 genes whose expression is most strongly recruited by SNAP-a (as defined by the gene loadings on this latent factor, and reflecting the fraction of their single-cell expression variance that is explained by the latent factor or cell state). We first asked whether these 500 genes are enriched for strong (genome-wide significant) associations to common and rare variants in schizophrenia. These SNAP-a-defined genes were 14 times more likely (than other protein-coding genes) to reside at genomic loci implicated by common genetic variation in schizophrenia ($p = 5 \times 10^{-25}$, 95% confidence interval: 8.7-24, by logistic regression, based on this SNAP-a-500 gene set containing 26 of the 98 protein-coding genes at 105 loci at which associated haplotypes involved SNPs in just 1-2 genes). These genes were also 7 times more likely (than other protein-coding genes) to have strong evidence from rare variants in schizophrenia (95% CI: 2.3–21, $p = 5 \times 10^{-4}$, by logistic regression, based on the SNAP-a-500 gene set containing 4 of the 32 genes implicated at FDR<0.05 by the SCHEMA Consortium or by rare, intragenic deletions). Note that SNAP-a was significant even in models in which "preferential expression in neurons" was a competing predictive factor:

Genes with common variation implicated in schizophrenia (loci at which associated haplotypes involved SNPs in just 1-2 genes): prediction by logistic regressions using cell-identity and/or cellular-program (SNAP) membership:

	Estimate	Std. Error	z value	Pr(>|z|)
(Intercept)	-5.511932	0.1388874	-39.6863400	0.000000e+00
genes w/ expression most specific to astrocytes	0.134320	0.3412139	0.3936534	6.938370e-01
genes w/ expression most specific to neurons	1.323195	0.2280745	5.8015912	**6.568853e-09**

	Estimate	Std. Error	z value	Pr(>|z|)
(Intercept)	-5.7144114	0.1482871	-38.536128	0.000000e+00
genes w/ expression most specific to astrocytes	-0.5614668	0.3633355	-1.545312	1.222708e-01
genes w/ expression most specific to neurons	1.2475955	0.2320574	5.376238	**7.605827e-08**
genes associated with SNAP-a in astrocytes	2.6729857	0.2584410	10.342732	**4.514813e-25**

Genes with rare variation implicated in schizophrenia (SCHEMA FDR<0.05 + *NRXN1*): prediction by logistic regression using cell-identity and cellular-program (SNAP) membership:

	Estimate	Std. Error	z value	Pr(>|z|)
(Intercept)	-6.5952346	0.2363882	-27.900012	2.667152e-171
genes w/ expression most specific to astrocytes	-0.8651288	0.7686021	-1.125587	2.603402e-01
genes w/ expression most specific to neurons	0.6479477	0.4681928	1.383933	1.663788e-01
**genes associated with SNAP-a in astrocytes**	**1.9568408**	**0.5652152**	**3.462116**	**5.359452e-04**

In the above analysis of genes implicated by rare variants, baseline expression in neurons was not significant. The implication of neuronally-expressed genes in the study by the SCHEMA Consortium used a different type of analysis, which used a Wilcoxon rank-sum test to evaluate whether SCHEMA genes had higher levels of neuron-preferential expression than other protein-coding genes did. In that analysis, in Figure S17 of the SCHEMA paper [2], about a third of the

neuronal types tested yielded p-values less than 0.05, whereas no non-neuronal cell types did. We repeated this analysis with the data from the current study, with several subtypes of neurons yielding nominally significant results ($p<0.05$) but not astrocytes ($p=0.64$), in accordance with the earlier findings. Applying an analogous analysis to the gene loadings for SNAP-a indicated that genes implicated by rare variants in schizophrenia had higher (more positive) SNAP-a loadings ($p = 8 \times 10^{-5}$, Wilcoxon rank-sum test).

Cellular-program (SNAP) gene expression and schizophrenia genetics: genome-wide genetic association statistics

To evaluate, beyond these top genetic associations, whether common genetic variation in the genes recruited by SNAP-a contributes more broadly to schizophrenia risk, we further utilized the gene-level association statistics provided by MAGMA analysis [1,4], which evaluates, for every gene, the tendency of common patterns of genetic variation (as identified by principal components analysis) to have elevated levels of association. To integrate across these more-subtle genomic signals, we also used a larger number of genes prioritized by SNAP-a.

Analysis indicated that the 2,000 genes whose expression was most strongly recruited by SNAP-a had elevated MAGMA z-scores for association to schizophrenia ($p < 2 \times 10^{-20}$), while astrocyte-identity gene expression did not ($p = 0.53$).

Linear regression of genes' levels of schizophrenia association (MAGMA z-scores) against their membership in cell-type-specific expression and/or cell-program (SNAP) groups (top 2,000 genes)

	Estimate	Std. Error	t value	Pr(>|t|)
(Intercept)	1.04298801	0.01349453	77.2896942	0.000000e+00
genes w/ expression most specific to astrocytes	-0.02363672	0.03795977	-0.6226781	5.335046e-01
**genes associated with SNAP-a in astrocytes**	**0.35203328**	**0.03795977**	**9.2738519**	**2.010865e-20**

Since the number of genes in the SNAP-a gene set is a somewhat arbitrary parameter of this analysis, we explored the relationship of this enrichment to the gene depth (on the SNAP-a-ranked gene list) used in analysis. The results for eight gene depths are summarized in the table below. Genetic signals were most strongly concentrated at the top of the SNAP-a gene list (as seen by the regression coefficient estimate, first column); however, concentration was still present at greater gene depths, and the statistical significance of the enrichment (as estimated by the test statistic, third column) increased in more-inclusive analyses up to about 2,000 genes, at which point it began to drop.

**Gene depth used**	**Estimate**	Std. Error	**t value**	Pr(>|t|)
**100**	**1.02480691**	0.15924204	6.4355299	1.264059e-10
200	0.86780224	0.11293191	7.6842959	1.623806e-14
400	0.61147742	0.08089709	7.5587070	4.282410e-14
1000	0.44671707	0.05218176	8.5607889	1.217565e-17
**2000**	0.35203328	0.03795977	**9.2738519**	**2.010865e-20**
3000	0.26788176	0.03200028	8.3712313	6.150143e-17
4000	0.18779209	0.02875229	6.5313782	6.705553e-11
8000	0.04179646	0.02440499	1.7126192	0.08680125

This relationship can also be recognized visually in a plot of MAGMA z-score vs. genes ordered by their SNAP-a gene loadings, which suggests that enrichment is strongest among the genes ranked most highly by SNAP-a (far left on plot) gene list but that enrichment continues, albeit more modestly, over the top 2,000 or so genes.

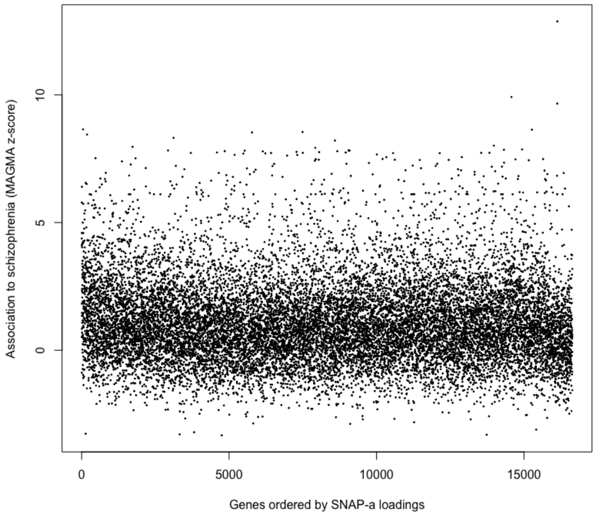

We also included neuronal-identity gene expression (as defined by the method used in the earlier studies) and SNAP-n-recruited genes in the regression analysis, as independent and competing predictive factors. All three were significant in a joint analysis, and the signal for SNAP-a genes was not attenuated by the inclusion of the two neuronal gene sets:

Linear regression of genes' levels of schizophrenia association (MAGMA z-scores) against their membership in cell-type-specific expression and/or cell-program (SNAP) groups (top 2,000 genes)

	Estimate	Std. Error	t value	Pr(>|t|)
(Intercept)	1.01002575	0.01469833	68.7170433	0.000000e+00
genes w/ expression most specific to astrocytes	-0.01596758	0.03819846	-0.4180164	6.759406e-01
genes w/ expression most specific to neurons	0.15147290	0.03767309	4.0207191	**5.827665e-05**
genes associated with SNAP-a in astrocytes	0.33732706	0.03807661	8.8591666	**8.861497e-19**
**genes associated with SNAP-n in neurons**	**0.14417190**	**0.03947272**	**3.6524436**	**2.605539e-04**

In the above result, both SNAP-n genes and neuronally-preferentially-expressed genes contributed to explaining gene-schizophrenia associations (MAGMA z-statistics), suggesting that – in neurons as in astrocytes – information about dynamic gene-expression programs can provide additional information beyond the information provided by cell-identity gene expression.

Finally, we used LD score regression [5] to evaluate per-SNP heritability enrichment across 27 brain phenotypes. Baseline astrocyte-identity gene expression (top 2,000 genes) did not exhibit heritability enrichment for any of the 26 brain phenotypes tested **(Supplementary Fig. 12a)**. SNAP-a (most strongly recruited 2,000 genes) exhibited per-SNP heritability enrichment ($p = 4 \times 10^{-5}$) for schizophrenia, nominal significance ($p < 0.01$) for smoking cessation and autism, and was not significant for the other 23 phenotypes tested **(Supplementary Fig. 12b)**.

References

1. Trubetskoy, V. *et al.* Mapping genomic loci implicates genes and synaptic biology in schizophrenia. *Nature* 2020.09.12.20192922 (2022).
2. Singh, T. *et al.* Rare coding variants in ten genes confer substantial risk for schizophrenia. *Nature* 2020.09.18.20192815 (2022).
3. Skene, N. G. *et al.* Genetic identification of brain cell types underlying schizophrenia. *Nat. Genet.* **50**, 825–833 (2018).
4. de Leeuw, C. A., Mooij, J. M., Heskes, T. & Posthuma, D. MAGMA: generalized gene-set analysis of GWAS data. *PLoS Comput. Biol.* **11**, e1004219 (2015).
5. Finucane, H. K. *et al.* Partitioning heritability by functional annotation using genome-wide association summary statistics. *Nat. Genet.* **47**, 1228–1235 (2015).

SUPPLEMENTARY TABLES

Supplementary Table 1. Summary of human tissue donor metadata.
Donor metadata table. Sample details include sex, age, post-mortem interval (PMI, when available), schizophrenia case-control status, and inclusion in experimental batches.

Supplementary Table 2. Donor expression levels and gene loadings for latent factors.
Tables of donor expression levels and genes-by-cell-types loadings for each of the 10 latent factors inferred by PEER.

Supplementary Table 3. Regression analysis of LF4 donor expression levels.
Joint regression analysis of LF4 donor expression levels with age, sex, and schizophrenia case-control status as independent variables.

Supplementary Table 4. Gene set enrichment analysis (GSEA) results for LF4 by cell type.
Tables of gene sets enriched in each cell type's component of LF4 (at FDR < 0.05) from a preranked gene set enrichment analysis (GSEA) using LF4 gene loadings.

Supplementary Table 5. Gene set enrichment analysis (GSEA) results for latent factors enriched in astrocytes.
Tables of gene sets enriched in astrocyte latent factors discovered by cNMF (at FDR < 0.15) from a preranked gene set enrichment analysis (GSEA) using gene loadings for each factor.

Supplementary Table 6. Donor expression levels and gene loadings for SNAP-a.
Tables of donor expression levels (mean cell scores by donor) and gene loadings for SNAP-a (astrocyte latent factor 2 inferred by cNMF).

Supplementary Table 7. Donor expression levels and gene loadings for SNAP-n.
Tables of donor expression levels (mean cell scores by donor) and gene loadings for SNAP-n (L5 IT glutamatergic neuron latent factor 6 inferred by cNMF).

Supplementary Table 8. Gene set enrichment analysis (GSEA) results for SNAP-n.
Table of gene sets enriched in SNAP-n (at FDR < 0.15) from a preranked gene set enrichment analysis (GSEA) using gene loadings for SNAP-n.

Supplementary Table 9. Genes in selected gene sets.
Table of genes in selected gene sets used in analyses. Descriptions of selected gene sets are in Methods.

SUPPLEMENTARY FIGURES

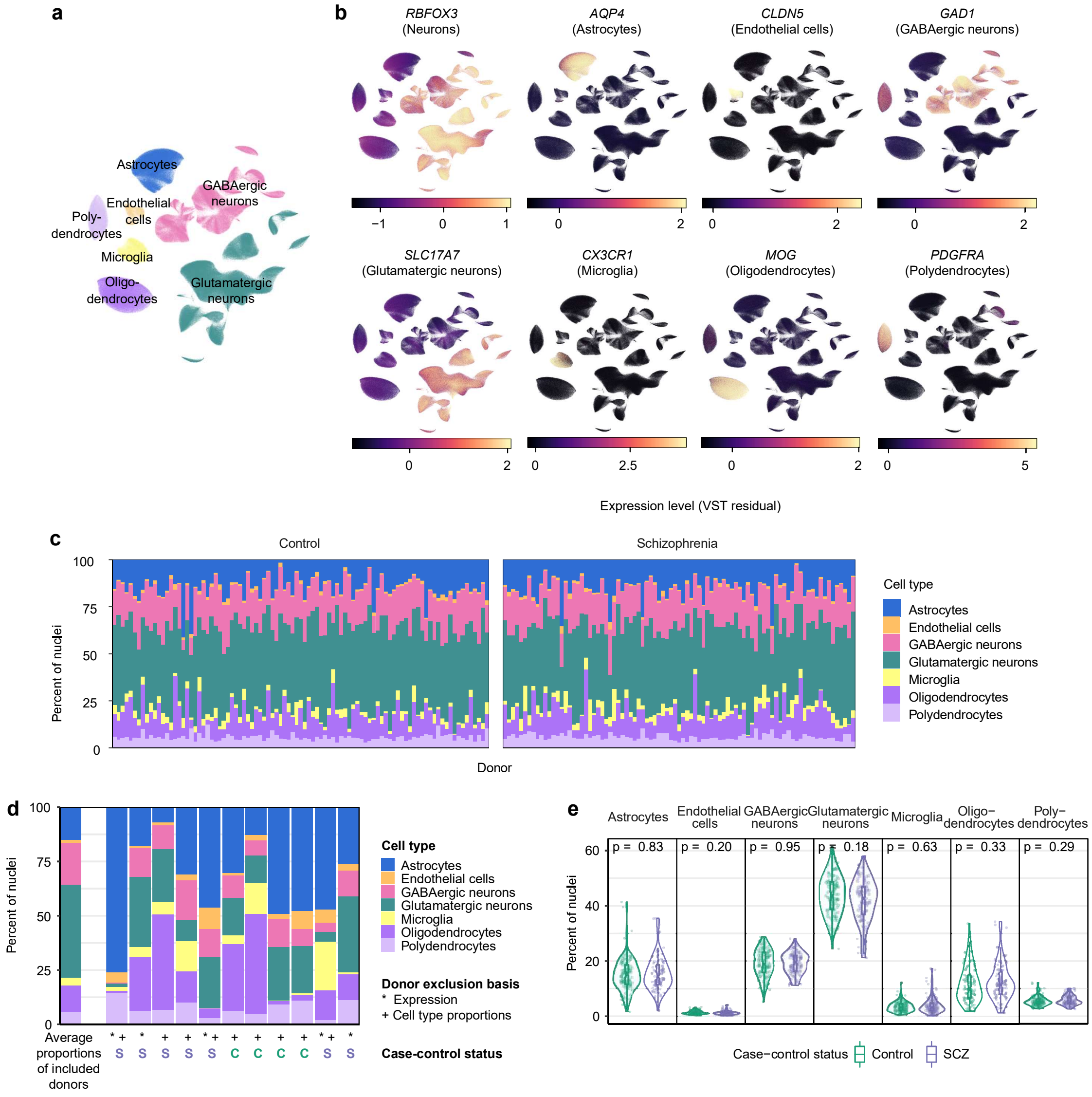

Supplementary Fig. 1 | Cell-type classification and composition analysis. a, Two-dimensional projection of the RNA-expression profiles of the 1,217,965 nuclei analyzed from 191 donors, reproduced from Fig. 1c. Nuclei are colored by their assignments to the major cell types present in Brodmann area 46 (BA46). The same projection is used in panel b. **b**, Expression levels of canonical marker genes of cell types in BA46. Values represent Pearson residuals from variance stabilizing transformation (VST). **c**, Relative representation of each cell type among nuclei ascertained from each donor. Donors are ordered by their anonymized research IDs at the Harvard Brain Tissue Resource Center. **d**, Cell-type proportions detected in 11 donors whom we excluded from subsequent analyses, with the basis of exclusion (unusual cell-type proportions and/or expression profiles) indicated under each donor. For comparison, average cell-type proportions of the 180 donors included in subsequent analyses are displayed to the left (donors from panel c). **e**, Cell-type proportions ascertained in the BA46 tissue samples; data points are separated by schizophrenia status (n = 93 unaffected and 87 affected). P-values from a two-sided Wilcoxon rank-sum test comparing the affected to the unaffected donors are reported at the top of each panel. Box plots show interquartile ranges; whiskers, 1.5x the interquartile interval; central lines, medians; notches, confidence intervals around medians.

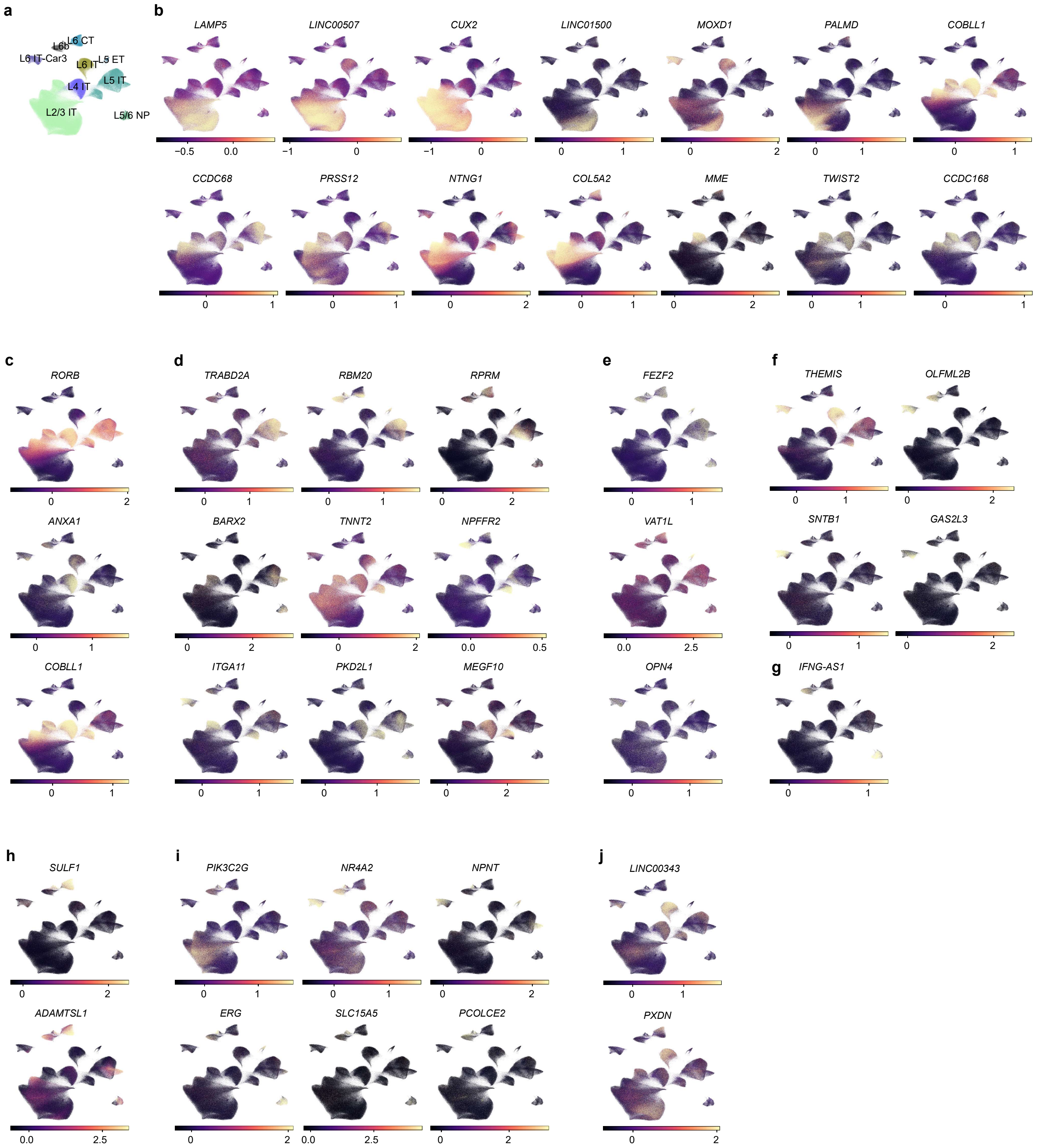

Supplementary Fig. 2 | Expression of glutamatergic neuron-subtype marker genes. a, Two-dimensional projection of the RNA-expression profiles of 524,186 glutamatergic neuron nuclei, reproduced from Fig. 1d. Nuclei are colored by their assignments to subtypes of glutamatergic neurons using classifications from [75] and [76]. The same projection is used in panels b to j below. **b-j**, Expression levels of marker genes for subtypes of **(b)** L2/3 IT, **(c)** L4 IT, **(d)** L5 IT, **(e)** L5 ET, **(f)** L6 IT-Car3, **(g)** L5/6 NP, **(h)** L6 CT, **(i)** L6b, and **(j)** L6 IT glutamatergic neurons. Markers are from [75] or from transcriptomically similar subtypes in [76]. Values represent Pearson residuals from variance stabilizing transformation (VST).

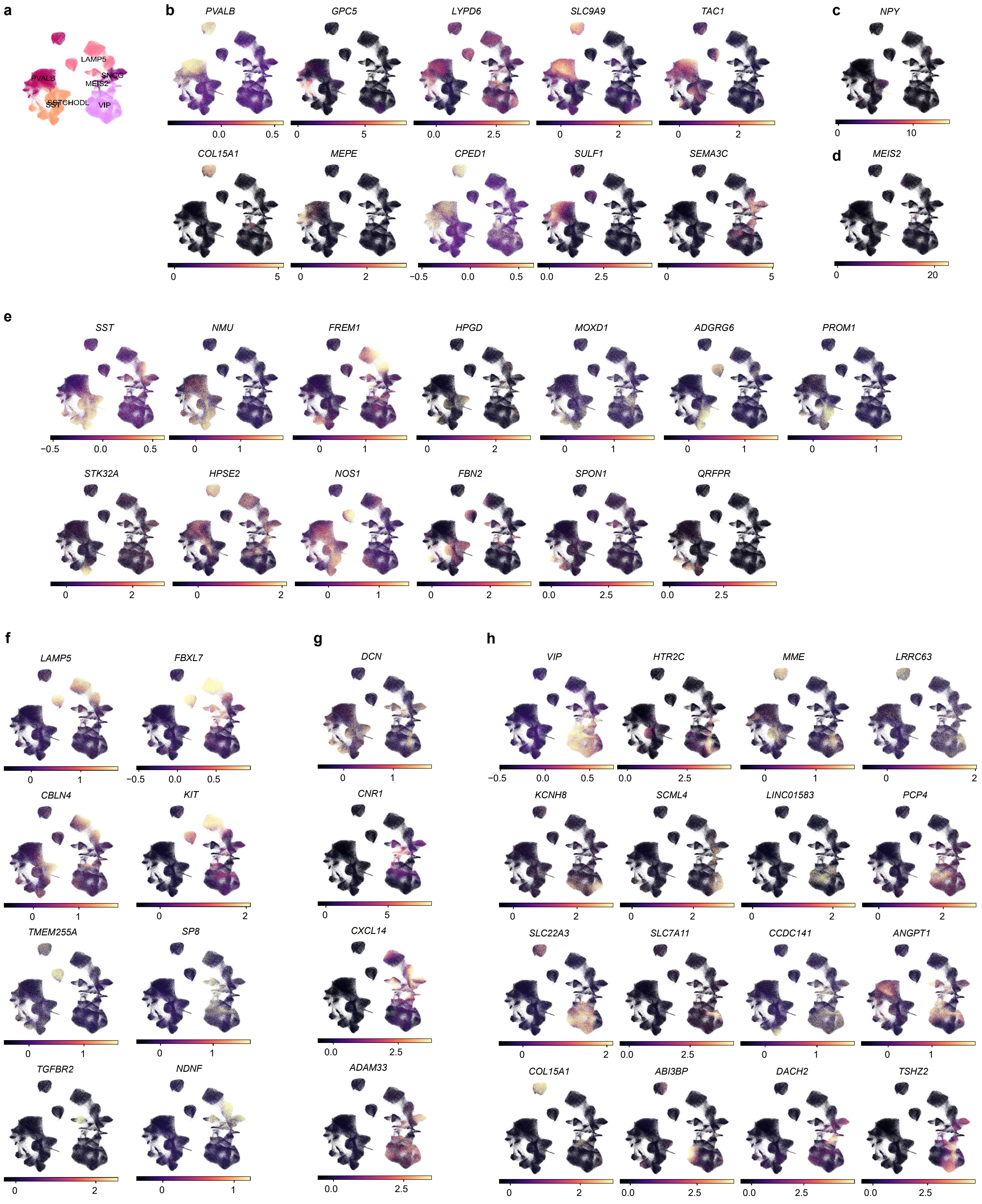

Supplementary Fig. 3 | Expression of GABAergic neuron-subtype marker genes. a, Two-dimensional projection of the RNA-expression profiles of 238,311 GABAergic neuron nuclei, reproduced from Fig. 1e. Nuclei are colored by their assignments to subtypes of GABAergic neurons using classifications from [75] and [76]. The same projection is used in panels b to h. **b-h**, Expression levels of marker genes for subtypes of **(b)** PVALB, **(c)** SST-CHODL, **(d)** MEIS2, **(e)** SST, **(f)** LAMP5, **(g)** SNCG, and **(h)** VIP GABAergic neurons. Markers are from [75] or from transcriptomically similar subtypes in [76]. Values represent Pearson residuals from variance stabilizing transformation (VST).

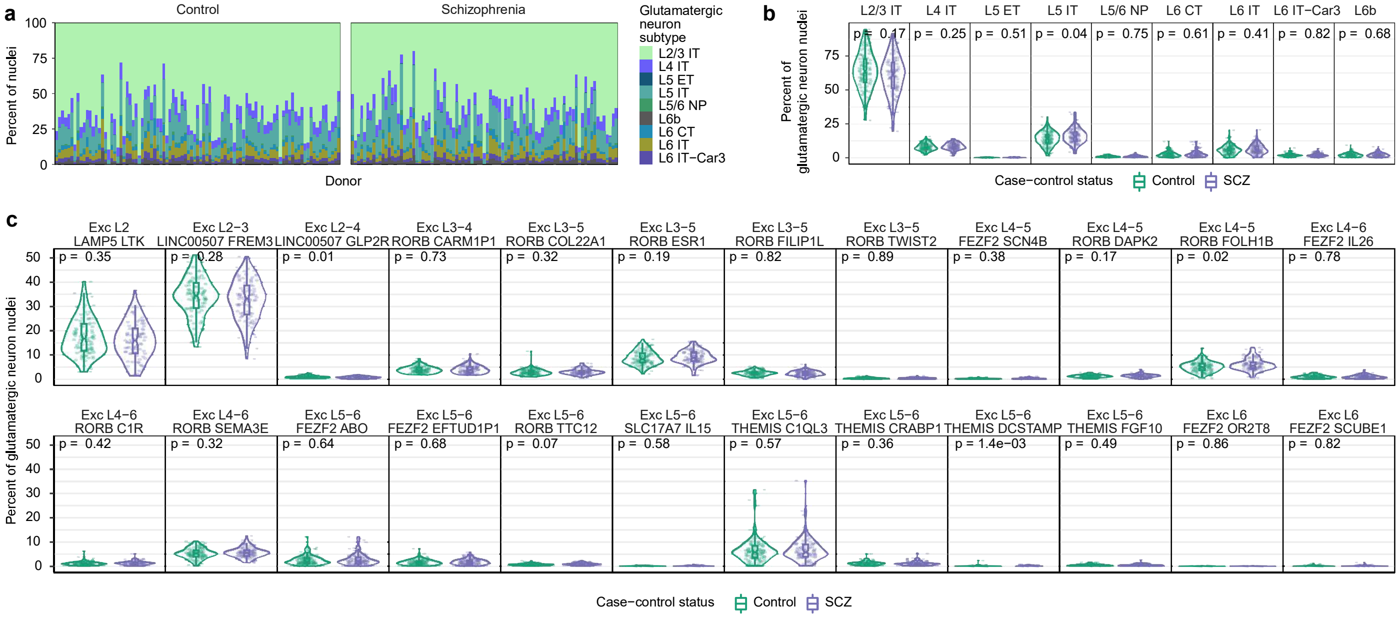

Supplementary Fig. 4 | Glutamatergic neuron-subtype composition analysis across donors. a, Relative representation of each glutamatergic neuron subtype among nuclei ascertained from each donor. Donors are ordered by their anonymized research IDs at the Harvard Brain Tissue Resource Center. **b-c**, Proportions of **(b)** glutamatergic neuron subtypes and **(c)** subtypes of these subtypes (defined in [75]) by schizophrenia status (n = 93 unaffected and 87 affected). P-values from a two-sided Wilcoxon rank-sum test comparing the affected to the unaffected donors are reported at the top of each panel. Box plots show interquartile ranges; whiskers, 1.5x the interquartile interval; central lines, medians; notches, confidence intervals around medians.

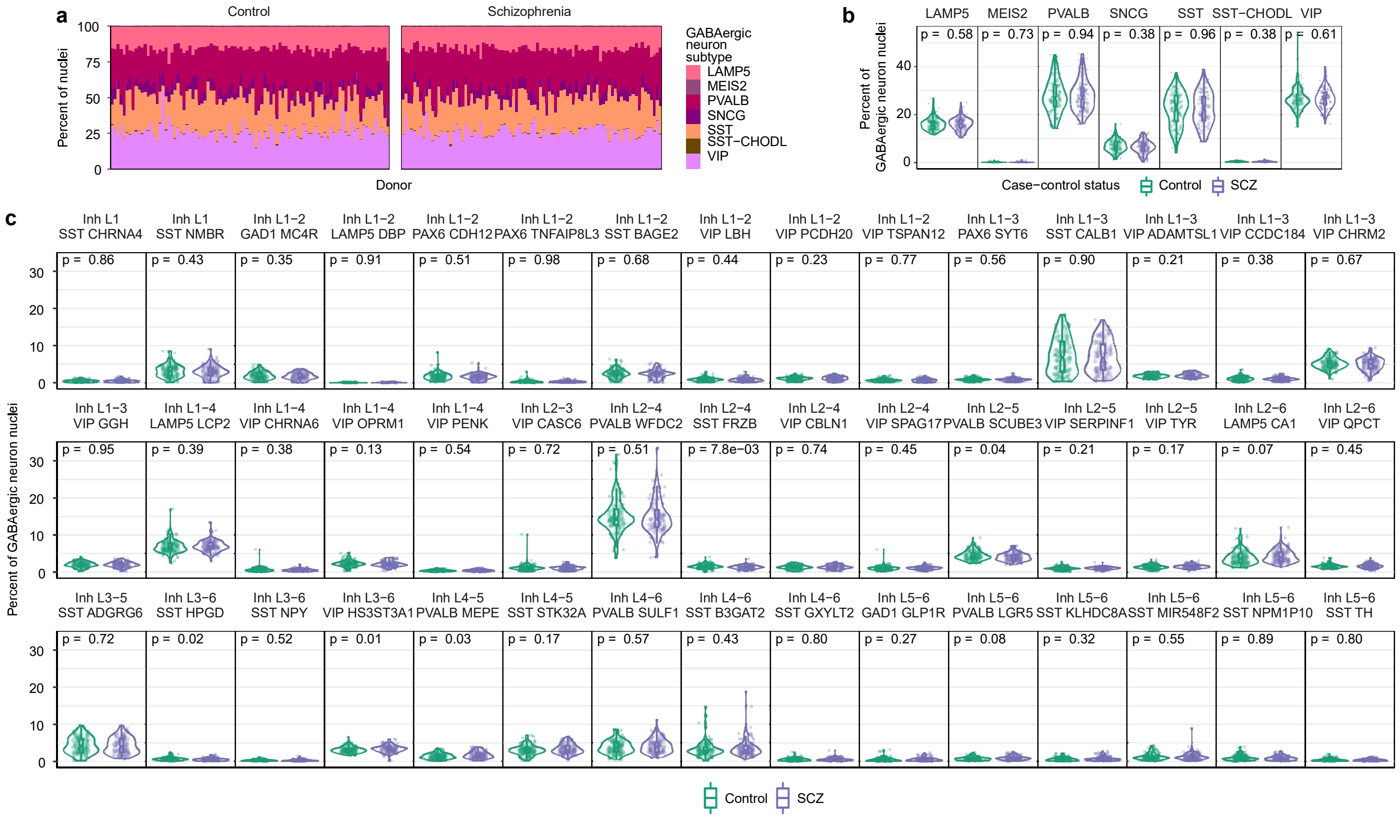

Supplementary Fig. 5 | GABAergic neuron-subtype composition analysis across donors. a, Relative representation of each GABAergic neuron subtype among nuclei ascertained from each donor. Donors are ordered by their anonymized research IDs at the Harvard Brain Tissue Resource Center. **b-c**, Proportions of **(b)** GABAergic neuron subtypes and **(c)** subtypes of these subtypes (defined in [75]) by schizophrenia status (n = 93 unaffected and 87 affected). P-values from a two-sided Wilcoxon rank-sum test comparing the affected to the unaffected donors are reported at the top of each panel. Box plots show interquartile ranges; whiskers, 1.5x the interquartile interval; central lines, medians; notches, confidence intervals around medians.

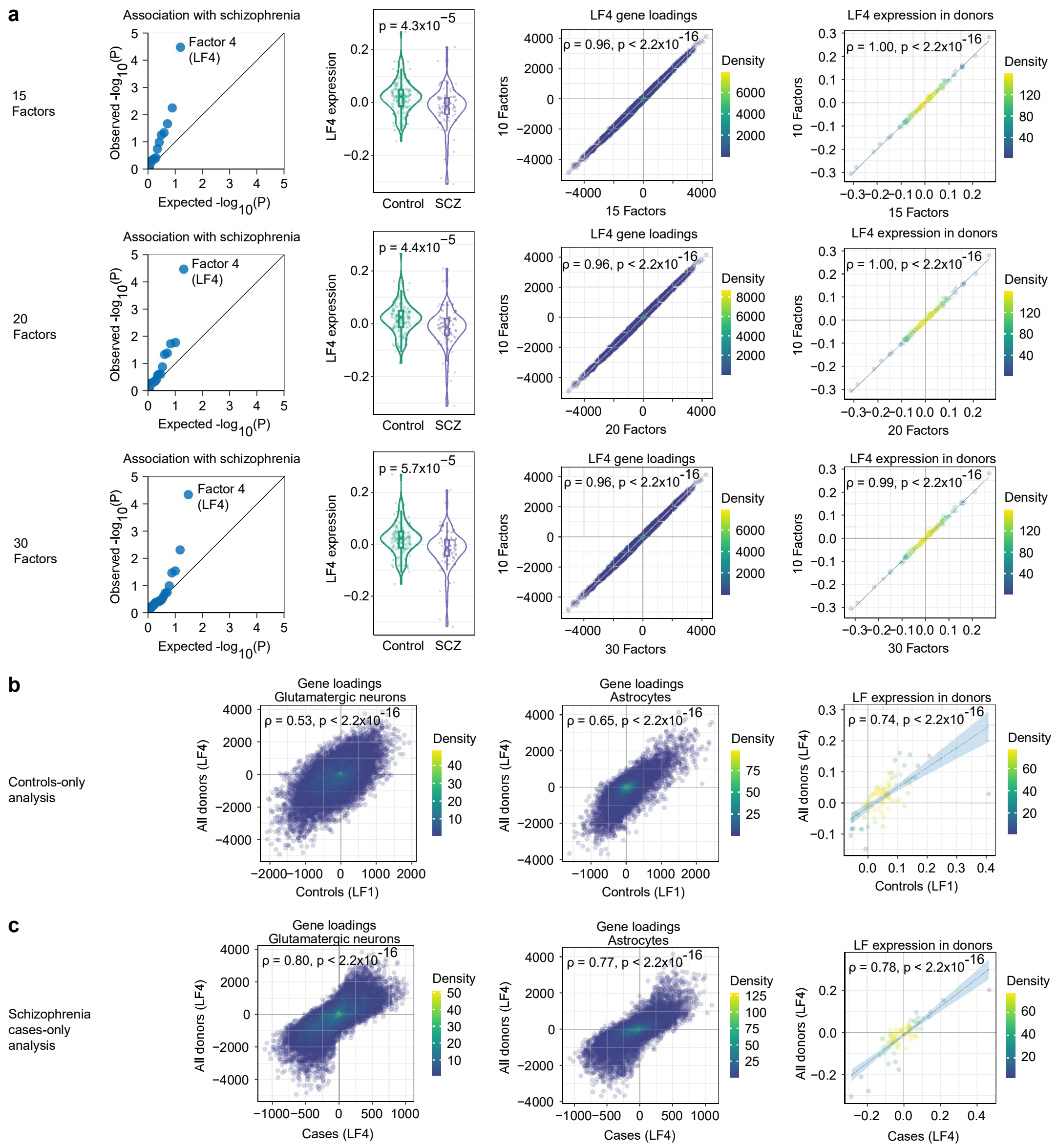

Supplementary Fig. 6 | Robustness of Latent Factor 4 (LF4) to analysis parameters. LFs similar to LF4 were identified in **(a)** analyses with different numbers of factors (*n* = 180 donors), **(b)** a controls-only analysis (*n* = 93 donors), and **(c)** a cases-only analysis (*n* = 87 donors). **a**, Column 1: Association of latent-factor expression levels with schizophrenia case-control status, shown as a quantile-quantile plot that compares observed -$\log_{10}$ *p*-values to the distribution of -$\log_{10}$ *p*-values expected under a null hypothesis (*n* = 15, 20, and 30 factors). The observed *p*-values were calculated for each latent factor by a two-sided Wilcoxon rank-sum test of latent factor expression levels (by donor) between cases and controls. In all analyses, LF4 is the factor that deviates the most from the line of unity and displays the strongest association with schizophrenia case-control status. Column 2: Expression of LF4 by case-control status (*n* = 93 controls and 87 cases). *P*-values are from a two-sided Wilcoxon rank-sum test. Box plots show interquartile ranges; whiskers, 1.5x the interquartile interval; central lines, medians; notches, confidence intervals around medians. Shaded regions represent 95% confidence intervals. Column 3: Comparison of gene loadings (*n* = 125,437 gene/cell-type combinations) that demonstrates the relationship of LF4 inferred from an analysis requesting 10 factors to LF4 inferred from an analysis requesting 15, 20, or 30 factors (Spearman's ρ). Shaded regions around regression lines represent 95% confidence intervals. Column 4: Comparison of donor expression levels (*n* = 180 donors) that demonstrates the relationship of LF4 inferred from an analysis requesting 10 factors to LF4 inferred from an analysis requesting 15, 20, or 30 factors (Spearman's ρ). Shaded regions around regression lines represent 95% confidence intervals. **b**, Column 1: Comparison of gene loadings from glutamatergic neurons (*n* = 18,829 genes) that demonstrates the relationship of LF4 inferred from an analysis of all donors to LF1 inferred from an analysis of only control donors (Spearman's ρ). Shaded regions around regression lines represent 95% confidence intervals. Column 2: Similar plot as in Column 1, here plotting gene loadings from astrocytes (*n* = 18,346 genes). Column 3: Comparison of donor expression levels (*n* = 180 donors) that demonstrates the relationship of LF4 inferred from an analysis of all donors to LF1 inferred from an analysis of only control donors (Spearman's ρ). Shaded regions around regression lines represent 95% confidence intervals. **c**, Similar plots as in b, here for the relationship of LF4 inferred from an analysis of all donors to LF4 inferred from an analysis of only donors with schizophrenia.

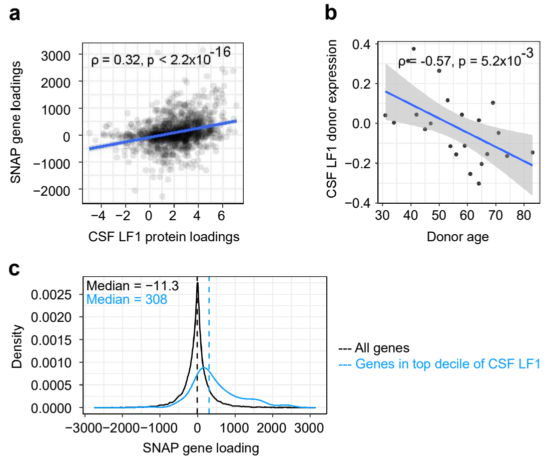

Supplementary Fig. 7 | Latent factor analysis of cerebrospinal fluid (CSF) proteomics data from different individuals identifies a factor resembling SNAP. To assess the biological significance of SNAP, we also sought evidence that SNAP manifests in the proteins that can be sampled from cerebrospinal fluid (CSF). We analyzed available data from a mass-spectrometry proteomics analysis of cerebrospinal fluid (CSF) from 22 healthy human donors [82], performing a latent factor analysis that is conceptually analogous to our analysis (in Fig. 1f) of cell-type-specific RNA-expression measurements in the brain donors (but of an independent data set, derived from a distinct set of donors). The top latent factor in analysis of the CSF proteomics data (explaining >15% of inter-individual variation in CSF protein measurements) bore a strong resemblance to SNAP. **a**, Relationship of SNAP gene loadings to the top latent factor in an analysis of inter-individual variation in CSF protein levels (CSF LF1) using quantitative protein abundance measurements from [82] (Spearman's ρ; *n* = 1,341 genes/proteins shared between both analyses). For SNAP, each gene is represented by a single composite loading representing gene loadings from all cell types (weighted by its median expression in each cell type). Shaded region represents 95% confidence interval. **b**, Relationship of CSF LF1 donor scores to age (Spearman's ρ; *n* = 22 donors). Shaded region represents 95% confidence interval. **c**, Density plot showing distribution of SNAP gene loadings for (black) all genes and genes encoding proteins that are strongly recruited (top decile) by (blue) CSF LF1. Distributions were found to be different by Wilcox test ($p = 2.1\times10^{-28}$, two-sided Wilcoxon rank-sum test).

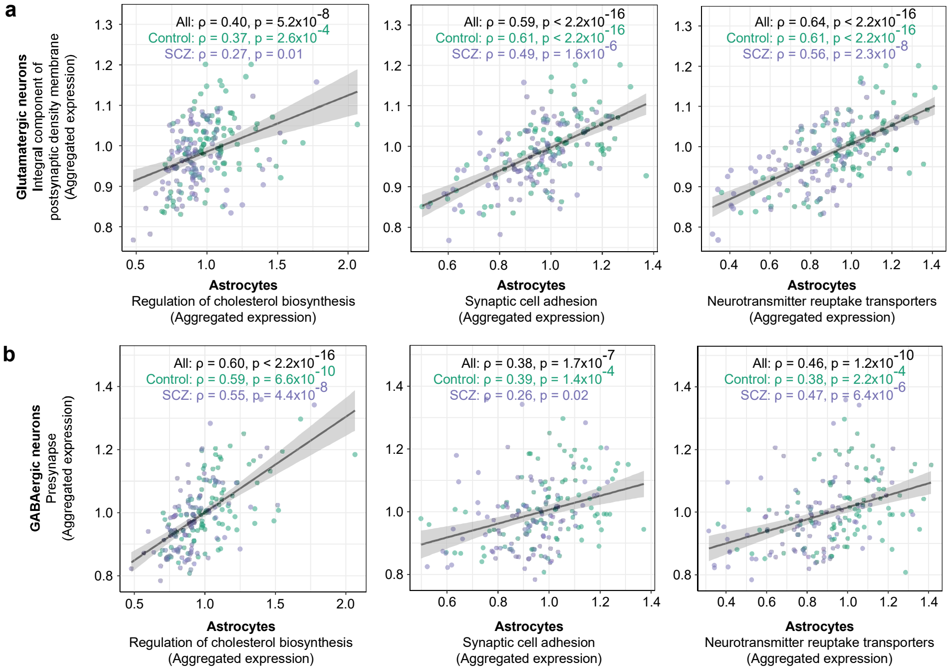

Supplementary Fig. 8 | Concerted synaptic investments by neurons and astrocytes. See also Fig. 2e. **a**, Relationship of donors' glutamatergic-neuron transcriptional investments in genes that are integral components of the postsynaptic density membrane (core genes contributing to the enrichment of GO:0099061) to astrocyte transcriptional investments in (top) cholesterol biosynthesis, (middle) synaptic adhesion, and (bottom) neurotransmitter reuptake transporters (Spearman's ρ). Expression values are the fraction of all UMIs in each donor (from the indicated cell type) that are derived from these genes, normalized to the median expression among control donors. Shaded regions represent 95% confidence intervals. **b**, Similar plots as in a, here for donors' GABAergic-neuron transcriptional investments in presynapse genes (core genes contributing to the enrichment of GO:0098793) on the x-axis.

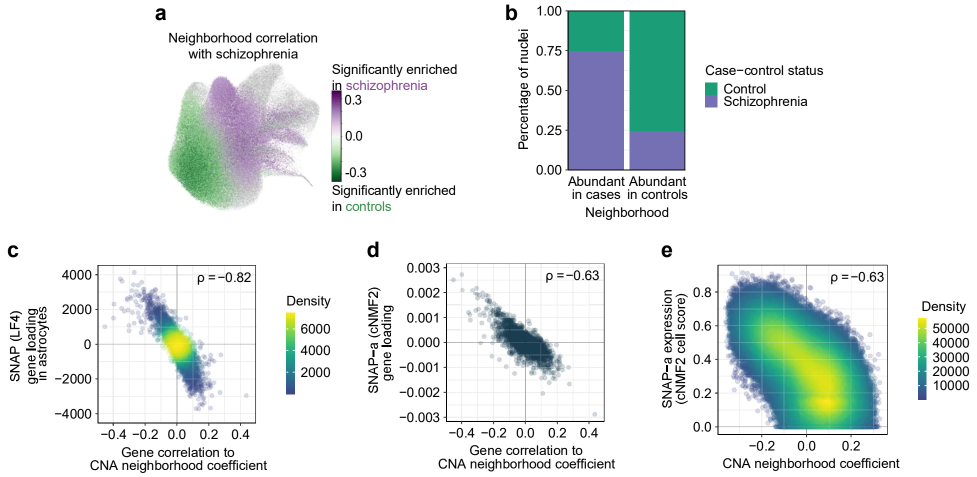

Supplementary Fig. 9 | Identification of astrocyte transcriptional neighborhoods associated with schizophrenia case-control status by co-varying neighborhood analysis. To further assess the robustness of the astrocyte gene-expression changes represented by SNAP and SNAP-a, we employed a third computational approach, co-varying neighborhood analysis (CNA) [96]. **a**, Same projection as in Fig. 3a-c, but with points colored according to their transcriptional neighborhood's correlation to schizophrenia case-control status (*n* = 179,764 astrocyte nuclei from 180 donors). Among cells whose neighborhood coefficients passed an FDR < 0.05 threshold for association, purple indicates high correlation to case status and green indicates high correlation to control status. All other cells with FDR > 0.05 for association are colored in gray. **b**, Proportion of nuclei in each of the indicated astrocyte transcriptional neighborhoods that are assigned to schizophrenia cases and controls (*n* = 34,271 nuclei abundant in cases and 38,327 nuclei abundant in controls). **c-d**, Relationship of genes' correlation to schizophrenia-associated transcriptional neighborhoods to **(c)** the astrocyte component of SNAP (*n* = 8,997 shared genes) and **(d)** SNAP-a by their gene loadings (*n* = 9,015 shared genes) (Spearman's ρ). Genes plotted are the subsets of protein-coding genes (with expression levels of at least 1 UMI per 10^5) that are shared between the indicated pairs of analyses. **e**, Relationship of cell-level neighborhood coefficients for schizophrenia-associated transcriptional neighborhoods to SNAP-a cell scores (Spearman's ρ; *n* = 179,764 astrocytes).

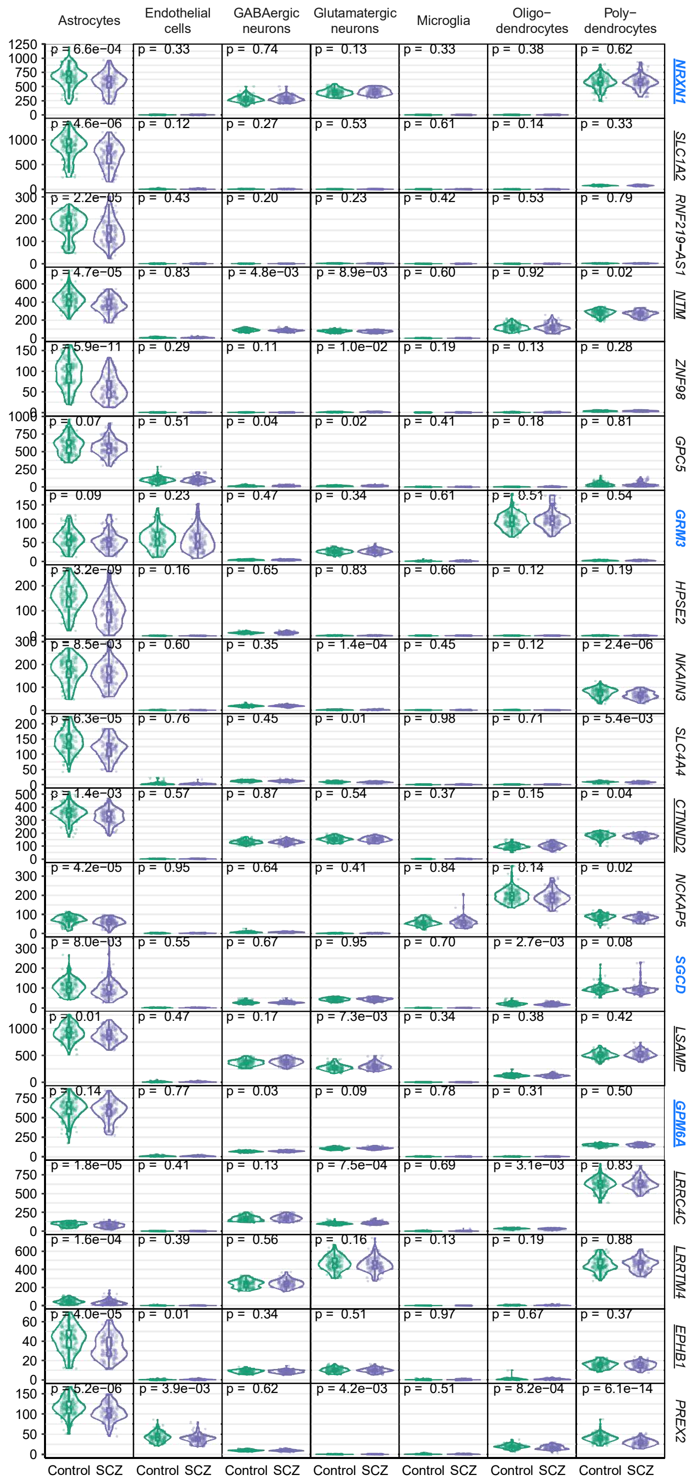

Supplementary Fig. 10 | Expression across cell types of genes most strongly recruited by SNAP-a. Expression in each cell type (by donor, separated by schizophrenia status), of the 20 genes that are most strongly recruited by SNAP-a (*n* = 93 unaffected (green) and 87 affected (purple) donors). These included eight genes with roles in adhesion of cells to synapses (*NRXN1*, *NTM*, *CTNND2*, *LSAMP*, *GPM6A*, *LRRC4C*, *LRRTM4*, and *EPHB1*) (as established by earlier work including [172–181] and reviewed in [11,12]). *P*-values from a two-sided Wilcoxon rank-sum test comparing the affected to the unaffected donors are reported at the top of each panel. Box plots show interquartile ranges; whiskers, 1.5x the interquartile interval; central lines, medians; notches, confidence intervals around medians. Genes that have been strongly implicated in human genetic studies of schizophrenia are highlighted in blue. Genes with known functions in synaptic adhesion (listed above) or neurotransmitter uptake (*SLC1A2*) are underlined.

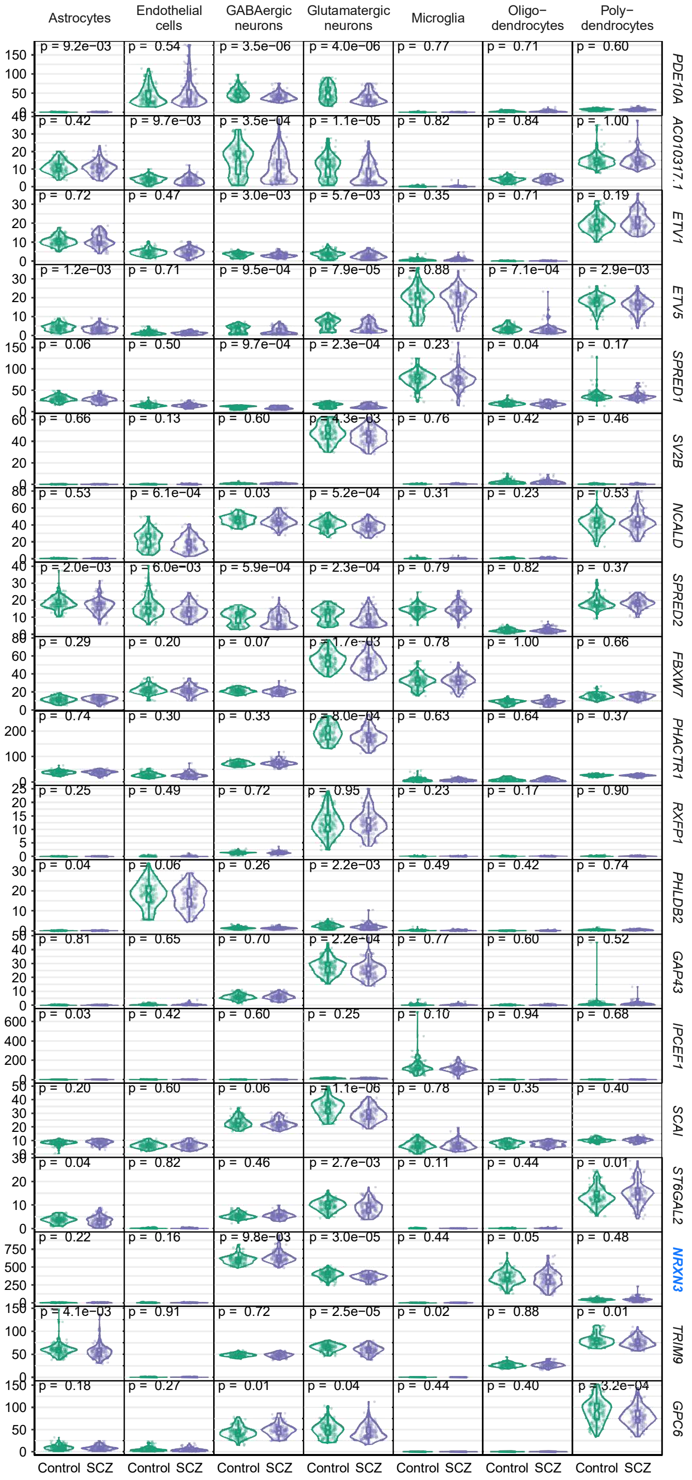

Supplementary Fig. 11 | Expression across cell types of genes most strongly recruited by SNAP-n. Expression in each cell type (by donor, separated by schizophrenia case-control status) of the 20 genes that are most strongly recruited by SNAP-n (*n* = 93 controls and 87 cases). *P*-values from a two-sided Wilcoxon rank-sum test comparing the affected to the unaffected donors are reported at the top of each panel. Box plots show interquartile ranges; whiskers, 1.5x the interquartile interval; central lines, medians; notches, confidence intervals around medians. Genes that have been strongly implicated in human genetic studies of schizophrenia are highlighted in blue.

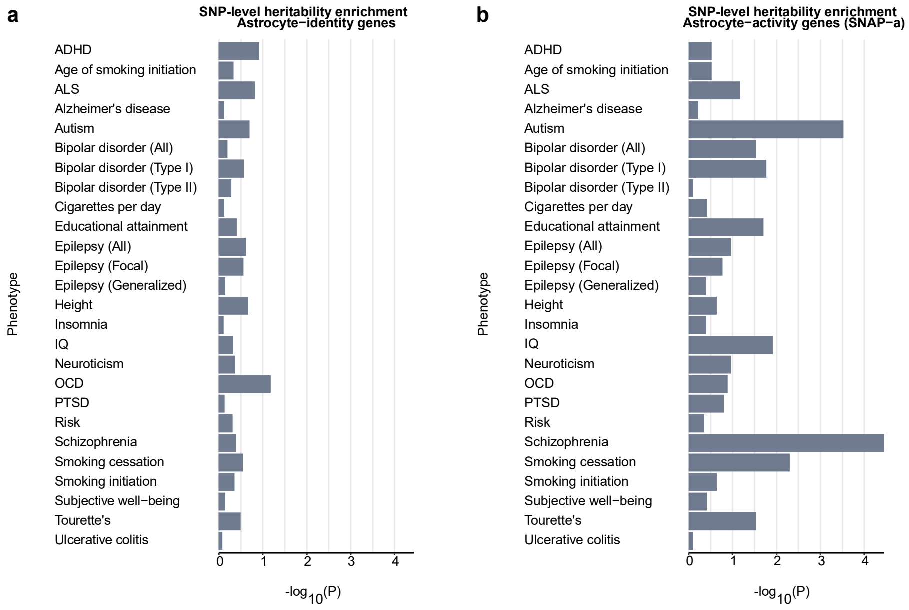

Supplementary Fig. 12 | Heritability enrichment for 26 traits among the top 2,000 astrocyte-identity or astrocyte-activity (SNAP-a) genes. Heritability enrichment analysis for the indicated phenotypes in regions surrounding **(a)** the 2,000 genes most preferentially expressed in astrocytes compared to other cell types or **(b)** the 2,000 genes most strongly recruited by SNAP-a in astrocytes. Summary statistics are from the following studies: ADHD [112], age of smoking initiation [115], ALS [113], Alzheimer's disease [114], autism [116], bipolar disorder (all, type I, and type II) [117], cigarettes per day [115], educational attainment [118], epilepsy (all, focal, generalized) [119], height [120], insomnia [122], IQ [121], neuroticism [123], OCD [124], PTSD [125], risk [126], schizophrenia [22], smoking cessation [115], smoking initiation [115], subjective well-being [127], Tourette's [128], ulcerative colitis [129].

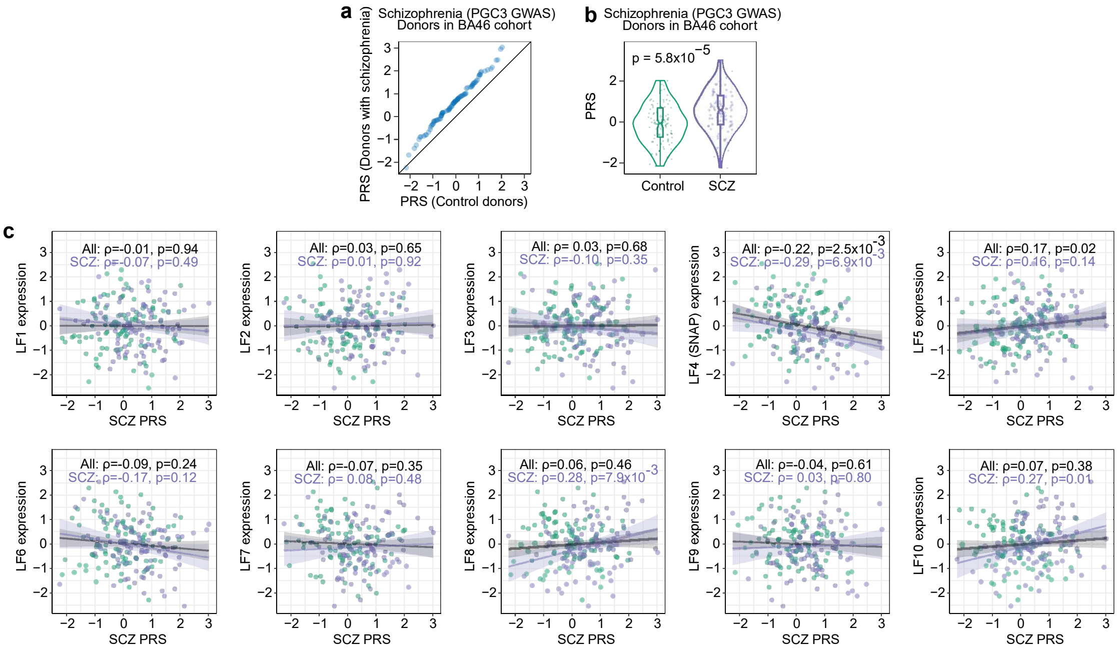

Supplementary Fig. 13 | Calculation of polygenic risk scores for schizophrenia. a, Association of polygenic risk scores (PRS) for schizophrenia (from PGC3 GWAS, [22]) with schizophrenia case-control status, displayed as a quantile-quantile plot that compares PRS of control donors to the PRS of donors with schizophrenia (*n* = 191 donors). **b**, Distributions of schizophrenia PRS for 94 schizophrenia cases and 97 controls. *P*-value is from a two-sided Wilcoxon rank-sum test. Box plots show interquartile ranges; whiskers, 1.5x the interquartile interval; central lines, medians; notches, confidence intervals around medians. **c**, See also Fig. 4b. Relationship of inter-individual variation in expression of each of the 10 latent factors inferred by PEER (donor scores, quantile-normalized) to donors' polygenic risk scores (PRSs) for schizophrenia (Spearman's ρ; PGC3 GWAS from [22]). Shaded regions represent 95% confidence intervals. The observed relationship of schizophrenia PRS to expression of LF4 – which associates with schizophrenia and aging – is consistent with previous observations that a PRS for schizophrenia also associates with decreased measures of cognition in older individuals [48] and with psychosis in Alzheimer's Disease [185].

[185] Creese, B. *et al.* Examining the association between genetic liability for schizophrenia and psychotic symptoms in Alzheimer's disease. *Transl. Psychiatry* **9**, 273 (2019).
