# Supplementary material for: A concerted neuron–astrocyte program declines in ageing and schizophrenia
Source: Nature. 2024 Mar 6;627(8004):604–11. doi: 10.1038/s41586-024-07109-5 (PMC10954558; doi:10.1038/s41586-024-07109-5)
Supplement: Supplementary file 2 — Reporting Summary [file 41586_2024_7109_MOESM2_ESM.pdf]

nature portfolio

Corresponding author(s): Steven A. McCarroll
Sabina Berretta
Emi Ling

Last updated by author(s): Dec 26, 2023

Reporting Summary

Nature Portfolio wishes to improve the reproducibility of the work that we publish. This form provides structure for consistency and transparency in reporting. For further information on Nature Portfolio policies, see our Editorial Policies and the Editorial Policy Checklist.

Statistics

For all statistical analyses, confirm that the following items are present in the figure legend, table legend, main text, or Methods section.

n/a	Confirmed	
☐	☒	The exact sample size (*n*) for each experimental group/condition, given as a discrete number and unit of measurement
☐	☒	A statement on whether measurements were taken from distinct samples or whether the same sample was measured repeatedly
☐	☒	The statistical test(s) used AND whether they are one- or two-sided<br>*Only common tests should be described solely by name; describe more complex techniques in the Methods section.*
☐	☒	A description of all covariates tested
☐	☒	A description of any assumptions or corrections, such as tests of normality and adjustment for multiple comparisons
☐	☒	A full description of the statistical parameters including central tendency (e.g. means) or other basic estimates (e.g. regression coefficient) AND variation (e.g. standard deviation) or associated estimates of uncertainty (e.g. confidence intervals)
☐	☒	For null hypothesis testing, the test statistic (e.g. *F*, *t*, *r*) with confidence intervals, effect sizes, degrees of freedom and *P* value noted<br>*Give P values as exact values whenever suitable.*
☒	☐	For Bayesian analysis, information on the choice of priors and Markov chain Monte Carlo settings
☒	☐	For hierarchical and complex designs, identification of the appropriate level for tests and full reporting of outcomes
☐	☒	Estimates of effect sizes (e.g. Cohen's *d*, Pearson's *r*), indicating how they were calculated

Our web collection on statistics for biologists contains articles on many of the points above.

Software and code

Policy information about availability of computer code

Data collection	Software and core computational analysis for the following data collection steps are freely available at https://github.com/broadinstitute/Drop-seq:<br>Drop-seq (v2.4.1) (Macosko et al. 2015) - align and process sequencing reads<br>Dropulation (v2.4.1) (Wells et al. 2023) - perform donor assignment
Data analysis	Software/tools/algorithms/packages used for data analyses are listed below and also cited in the text.<br>anndata (v0.8.0) (Virshup et al. 2021) - https://anndata.readthedocs.io<br>BBKNN (v1.5.1) (Polański et al. 2020) - https://github.com/Teichlab/bbknn<br>BCFtools (v1.16) (Danecek et al. 2021) - https://www.htslib.org<br>Bowtie2 (v2.2.4) (Langmead et al. 2012) - https://bowtie-bio.sourceforge.net/bowtie2<br>CellBender (v0.1.0) (Fleming et al. 2023) - https://github.com/broadinstitute/CellBender<br>cluster (v2.1.2) (Maechler et al. 2022) - https://CRAN.R-project.org/package=cluster<br>clustree (v0.4.4) (Zappia et al. 2018) - https://github.com/lazappi/clustree<br>CNA (v0.1.4) (Reshef et al. 2022) - https://github.com/immunogenomics/cna<br>cNMF (v1.2) (Kotliar et al. 2019) - https://github.com/dylkot/cNMF<br>Color Oracle (v1.3) (Jenny et al. 2006; Jenny et al. 2007) - https://github.com/nvkelso/color-oracle-java<br>ComplexHeatmap (v2.10.0) (Gu et al. 2016; Gu 2022) - https://github.com/jokergoo/ComplexHeatmap<br>data.table (v1.14.8) (Dowle et al. 2023) - https://CRAN.R-project.org/package=data.table<br>DescTools (v0.99.48) (Signorell 2023) - https://CRAN.R-project.org/package=DescTools<br>dplyr (v1.1.2) (Wickham et al. 2023) - https://CRAN.R-project.org/package=dplyr<br>fastICA (v1.2-1) (Marchini et al. 2017) - https://CRAN.R-project.org/package=fastICA

FUMA (v1.5.6) (Watanabe et al. 2017) - https://fuma.ctglab.nl
gdata (v2.19.0) (Warnes et al. 2023) - https://CRAN.R-project.org/package=gdata
GenCall (v3.0.0) (Kermani 2006)
Genome STRiP (v2.0) (Handsaker et al. 2015) - https://software.broadinstitute.org/software/genomestrip
ggforce (v0.4.1) (Pedersen 2022) - https://CRAN.R-project.org/package=ggforce
ggplot2 (v3.4.2) (Wickham 2016) - https://ggplot2.tidyverse.org
ggpmisc (v0.5.3) (Aphalo 2023) - https://CRAN.R-project.org/package=ggpmisc
ggpointdensity (v0.1.0) (Kremer 2019) - https://CRAN.R-project.org/package=ggpointdensity
ggpubr (v0.5.0) (Kassambara 2022) - https://CRAN.R-project.org/package=ggpubr
ggrastr (v1.0.2) (Petukhov et al. 2023) - https://CRAN.R-project.org/package=ggrastr
ggrepel (v0.9.3) (Slowikowski 2023) - https://CRAN.R-project.org/package=ggrepel
grid (v4.1.3) (R Core Team 2022) - https://www.R-project.org
gridExtra (v2.3) (Auguie 2017) - https://CRAN.R-project.org/package=gridExtra
GSEA (v4.0.3) (Subramanian et al. 2005; Mootha et al. 2003) - https://www.gsea-msigdb.org/gsea/index.jsp
gtable (v0.3.3) (Wickham et al. 2023) - https://CRAN.R-project.org/package=gtable
IMPUTE5 (v1.1.5) (Rubinacci et al. 2020) - https://jmarchini.org/software/#impute-5
lme4 (v1.1-31) (Bates et al. 2015) - https://github.com/lme4/lme4
MAGMA (v1.08) (de Leeuw et al. 2015) - http://ctglab.nl/software/magma
Matplotlib (v3.5.2) (Hunter et al. 2007) - https://matplotlib.org
matrixStats (v0.63.0) (Bengtsson 2022) - https://CRAN.R-project.org/package=matrixStats
minpack.lm (v1.2-4) (Elzhov et al. 2022) - https://CRAN.R-project.org/package=minpack.lm
MoChA WDL (v2022-12-21) (Loh et al. 2018; Loh et al. 2020) - https://github.com/freeseek/mochawdl
NumPy (v1.17.5) (Harris et al. 2020) - https://numpy.org
Osprey (v0.1-9) (Handsaker et al. 2022) - https://github.com/broadinstitute/Osprey
pandas (v1.0.5) (The pandas development team 2020; McKinney 2010) - https://pandas.pydata.org
PEER (v1.0) (Stegle et al. 2012) - https://github.com/PMBio/peer
pheatmap (v1.0.12) (Kolde 2019) - https://CRAN.R-project.org/package=pheatmap
plyr (v1.8.8) (Wickham 2011) - https://plyr.had.co.nz
presto (v1.0.0) (Korsunsky et al. 2022) - https://immunogenomics.github.io/presto
purrr (v1.0.1) (Wickham et al. 2023) - https://CRAN.R-project.org/package=purrr
pySCENIC (v0.11.2) (Aibar et al. 2017; Van de Sande et al. 2020) - https://github.com/aertslab/SCENICprotocol
RColorBrewer (v1.1-3) (Neuwirth 2022) - https://CRAN.R-project.org/package=RColorBrewer
readxl (v1.4.2) (Wickham et al. 2023) - https://CRAN.R-project.org/package=readxl
reshape2 (v1.4.4) (Wickham 2007) - https://github.com/hadley/reshape
ROSE (v1.3.1) (Whyte et al. 2013; Lin et al. 2013) - https://github.com/stjude/ROSE
S-LDSC (v1.0.1) (Finucane et al. 2015) - https://github.com/bulik/ldsc
samtools (v1.3.1) (Danecek et al. 2021) - https://www.htslib.org
scales (v1.2.1) (Wickham et al. 2023) - https://CRAN.R-project.org/package=scales
Scanpy (v1.9.1) (Wolf et al. 2018) - https://scanpy.readthedocs.io
score (v2022-12-21) (Loh et al. 2018; Loh et al. 2020) - https://github.com/freeseek/mochawdl
scPred (v1.9.2) (Alquicira-Hernandez et al. 2019) - https://github.com/powellgenomicslab/scPred
sctransform (v0.3.1) (Hafemeister et al. 2019) - https://github.com/satijalab/sctransform
seaborn (v0.10.1) (Waskom et al. 2021) - https://seaborn.pydata.org
Seurat (v3.2.2) (Stuart et al. 2019) - https://satijalab.org/seurat
SeuratDisk (v0.0.0.9010) (Hoffmann et al. 2022) - https://mojaveazure.github.io/seurat-disk
SHAPEIT4 (v4.2.2) (Delaneau et al. 2019) - https://github.com/odelaneau/shapeit4
splitstackshape (v1.4.8) (Mahto 2019) - https://CRAN.R-project.org/package=splitstackshape
stats (v4.1.3) (R Core Team 2022) - https://www.R-project.org
stringi (v1.7.12) (Gagolewski 2022) - https://stringi.gagolewski.com
stringr (v1.5.0) (Wickham 2022) - https://CRAN.R-project.org/package=stringr
tidyr (v1.3.0) (Wickham et al. 2023) - https://CRAN.R-project.org/package=tidyr
Trimmomatic (v0.33) (Bolger et al. 2014) - http://www.usadellab.org/cms/?page=trimmomatic
viridis (v0.6.2) (Garnier et al. 2021) - https://sjmgarnier.github.io/viridis

For manuscripts utilizing custom algorithms or software that are central to the research but not yet described in published literature, software must be made available to editors and reviewers. We strongly encourage code deposition in a community repository (e.g. GitHub). See the Nature Portfolio guidelines for submitting code & software for further information.

Data

Policy information about availability of data

All manuscripts must include a data availability statement. This statement should provide the following information, where applicable:

- Accession codes, unique identifiers, or web links for publicly available datasets
- A description of any restrictions on data availability
- For clinical datasets or third party data, please ensure that the statement adheres to our policy

Sequencing data generated in this study and processed sequencing files are available through the Neuroscience Multi-omic Data Archive (NeMO) (RRID:SCR_016152) at https://assets.nemoarchive.org/dat-bmx7s1t. The data are available under controlled use conditions set by human privacy regulations. To access the data, the requester must first create an account in DUOS (https://duos.broadinstitute.org) using their institutional email address. The Signing Official from the requester's institution must also register in DUOS to issue the requester a Library Card Agreement. The requester will then need to fill out a Data Access Request through DUOS, which will be reviewed by the Broad Institute's Data Access Committee. Once a request is processed, NeMO will be notified to authorize access to the data. Processed expression data can also be queried using an interactive public web interface that we created (https://sz.mccarrolllab.org/app/SZ). Source data with anonymized donor IDs are provided with this paper.

The following publicly available datasets were also analyzed: ProteomeXchange Dataset PXD026491 (Karayel et al. 2022) and Gene Expression Omnibus Series GSE147672 (Corces et al. 2020).

Research involving human participants, their data, or biological material

Policy information about studies with human participants or human data. See also policy information about sex, gender (identity/presentation), and sexual orientation and race, ethnicity and racism.

Reporting on sex and gender	Brain donors were selected for this study so that within every batch (containing 10 schizophrenia cases and 10 controls), every donor with schizophrenia had a sex- and age-matched control. Information relevant to consent for sharing of individual-level data is described under "Ethics oversight". Key findings from this paper apply to both sexes (i.e. SNAP, SNAP-a).
Reporting on race, ethnicity, or other socially relevant groupings	This study does not use socially constructed or socially relevant categorization variables.
Population characteristics	Brain tissue samples were obtained from 191 postmortem donors (97 controls, 94 schizophrenia cases). Median age, 64; median postmortem interval, 22.7 hours; no significant differences in these covariates between cases and controls.
Recruitment	Brain donors were recruited by the Harvard Brain Tissue Resource Center/NIH NeuroBioBank (HBTRC/NBB), in a community-based manner, across the USA. To minimize biases for this specific study, donors unaffected by nervous system disorders were selected as sex- and age-matched controls for donors with schizophrenia. Consensus diagnosis of schizophrenia was carried out by retrospective review of medical records and extensive questionnaires concerning social and medical history provided by family members. Several regions from each brain were examined by a neuropathologist. We excluded subjects with evidence for gross and/or macroscopic brain changes, or with clinical history consistent with cerebrovascular accident or other neurological disorders. Subjects with Braak stages III or higher (modified Bielchowsky stain) were not included. None of the subjects had significant history of substance dependence within 10 or more years from death, as further corroborated by negative toxicology reports. Absence of recent substance abuse is typical for samples from the HBTRC, which receives exclusively community-based tissue donations.
Ethics oversight	Human brain tissue was obtained from the HBTRC/NBB. The HBTRC procedures for informed consent by the donor's legal next-of-kin and distribution of de-identified postmortem tissue samples and demographic and clinical data for research purposes are approved by the Mass General Brigham Institutional Review Board. Post-mortem tissue collection followed the provisions of the United States Uniform Anatomical Gift Act of 2006 described in the California Health and Safety Code section 7150 and other applicable state and federal laws and regulations. Federal regulation 45 CFR 46 and associated guidance indicates that the generation of data from de-identified post-mortem specimens does not constitute human participant research that requires institutional review board review.

Note that full information on the approval of the study protocol must also be provided in the manuscript.

Field-specific reporting

Please select the one below that is the best fit for your research. If you are not sure, read the appropriate sections before making your selection.

☒ Life sciences ☐ Behavioural & social sciences ☐ Ecological, evolutionary & environmental sciences

For a reference copy of the document with all sections, see nature.com/documents/nr-reporting-summary-flat.pdf

Life sciences study design

All studies must disclose on these points even when the disclosure is negative.

Sample size	No sample size calculation was performed. Sample size was determined by the limited availability of donors per diagnosis who met the study criteria (as described in "Recruitment"); these sizes were sufficient as they are similar to or exceed sample sizes from published bulk RNA-seq studies on postmortem human brain tissue. We aimed to analyze at least 50 donors per group (schizophrenia cases and age/sex-matched controls) to account for potential drop-outs in the analysis pipeline, which could be due to factors such as poor tissue quality and genotyping issues that prevented assignment of nuclei to an expected donor.
Data exclusions	Singlet nuclei were excluded if they could not be confidently assigned to a single expected donor (at FDR < 0.05), if they strongly expressed markers of more than one cell type and grouped together in a distinct cluster in UMAP space, or were assigned to donors with gene-expression profiles and/or cell-type-proportions that were distinct from the other donors in the cohort. Additional details are described in Methods.
Replication	Replication of the single-nucleus RNA-seq experiments was not attempted due to the limited availability of donors per diagnosis. We attempted to validate our findings using an orthogonal approach, and found evidence that an analogous constellation of changes also manifests at a protein level in variation among different individuals' cerebrospinal fluid protein profiles (analysis of data from Karayel et al. 2022).
Randomization	Randomization was not used. Specimens were allocated into batches of 20 specimens per batch, ensuring that the same number of cases and age-matched controls (10 per diagnosis), and men and women (10 per sex) were included in each batch. Specimens from cases and age-

matched controls were also processed in alternating order within each batch.

Blinding

To ensure that batch compositions were balanced, investigators were not blinded to the batch allocation or processing order of each specimen (as described for "Randomization"). Researchers had access to unique numerical codes assigned to the donor-of-origin of each specimen as well as basic donor metadata (e.g. case-control status, age, sex).

Reporting for specific materials, systems and methods

We require information from authors about some types of materials, experimental systems and methods used in many studies. Here, indicate whether each material, system or method listed is relevant to your study. If you are not sure if a list item applies to your research, read the appropriate section before selecting a response.

Materials & experimental systems

n/a	Involved in the study
☒	☐ Antibodies
☒	☐ Eukaryotic cell lines
☒	☐ Palaeontology and archaeology
☒	☐ Animals and other organisms
☒	☐ Clinical data
☒	☐ Dual use research of concern
☒	☐ Plants

Methods

n/a	Involved in the study
☒	☐ ChIP-seq
☒	☐ Flow cytometry
☒	☐ MRI-based neuroimaging

Plants

Seed stocks

Report on the source of all seed stocks or other plant material used. If applicable, state the seed stock centre and catalogue number. If plant specimens were collected from the field, describe the collection location, date and sampling procedures.

Novel plant genotypes

Describe the methods by which all novel plant genotypes were produced. This includes those generated by transgenic approaches, gene editing, chemical/radiation-based mutagenesis and hybridization. For transgenic lines, describe the transformation method, the number of independent lines analyzed and the generation upon which experiments were performed. For gene-edited lines, describe the editor used, the endogenous sequence targeted for editing, the targeting guide RNA sequence (if applicable) and how the editor was applied.

Authentication

Describe any authentication procedures for each seed stock used or novel genotype generated. Describe any experiments used to assess the effect of a mutation and, where applicable, how potential secondary effects (e.g. second site T-DNA insertions, mosiacism, off-target gene editing) were examined.
